# Multiradionuclide evidence for the solar origin of the cosmic-ray events of ᴀᴅ 774/5 and 993/4

**DOI:** 10.1038/ncomms9611

**Published:** 2015-10-26

**Authors:** Florian Mekhaldi, Raimund Muscheler, Florian Adolphi, Ala Aldahan, Jürg Beer, Joseph R. McConnell, Göran Possnert, Michael Sigl, Anders Svensson, Hans-Arno Synal, Kees C. Welten, Thomas E. Woodruff

**Affiliations:** 1 Department of Geology—Quaternary Sciences, Lund University, 22362 Lund, Sweden; 2 Department of Geology, United Arab Emirates University, 17551 Al Ain, UAE; 3 Department of Earth Sciences, Uppsala University, 75236 Uppsala, Sweden; 4 Swiss Federal Institute of Aquatic Science and Technology, 8600 Dübendorf, Switzerland; 5 Division of Hydrologic Sciences, Desert Research Institute, Reno, Nevada 89512, USA; 6 Tandem Laboratory, Uppsala University, 75120 Uppsala, Sweden; 7 Laboratory for Radiochemistry and Environmental Chemistry, Paul Scherrer Institut, 5232 Villigen, Switzerland; 8 Center for Ice and Climate, Niels Bohr Institute, University of Copenhagen, 2100 Copenhagen, Denmark; 9 Laboratory of Ion Beam Physics, ETH Zürich, 8093 Zürich, Switzerland; 10 Space Sciences Laboratory, University of California, Berkeley, California 94720, USA; 11 PRIME Laboratory, Purdue University, West Lafayette, Indiana 47907, USA

## Abstract

The origin of two large peaks in the atmospheric radiocarbon (^14^C) concentration at AD 774/5 and 993/4 is still debated. There is consensus, however, that these features can only be explained by an increase in the atmospheric ^14^C production rate due to an extraterrestrial event. Here we provide evidence that these peaks were most likely produced by extreme solar events, based on several new annually resolved ^10^Be measurements from both Arctic and Antarctic ice cores. Using ice core ^36^Cl data in pair with ^10^Be, we further show that these solar events were characterized by a very hard energy spectrum with high fluxes of solar protons with energy above 100 MeV. These results imply that the larger of the two events (AD 774/5) was at least five times stronger than any instrumentally recorded solar event. Our findings highlight the importance of studying the possibility of severe solar energetic particle events.

## Abstract (web summary)

Natural spikes in radiocarbon have been identified at ᴀᴅ 774/5 and 993/4 and attributed to exceptional cosmic-ray events, although the cause remains uncertain. Here, the authors analyse records recovered from ice cores and suggest these spikes originated from extreme solar particle events.

## Graphical abstract

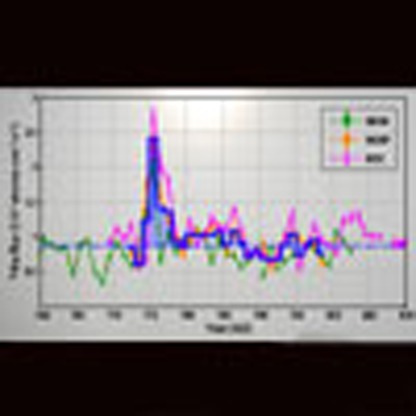

The sun irregularly expels large amounts of energetic particles into the interplanetary space and into the vicinity of the Earth which can be observed as so-called solar proton events (SPE). In the context of modern society, this poses a threat to communication, electronic and power systems^1^,^2^. In addition, SPEs are known to deplete ozone^3^,^4^ and thus possibly affect weather and atmospheric circulation^5^. In consequence, better assessing the relationship between magnitude and occurrence frequency of such events is of substantial importance for solar physics, space technologies, technological infrastructures and climate sciences. The largest known solar flare is considered to be the Carrington event of AD 1859 which is reported to have caused disturbances on telegraph systems and widespread auroral sightings^6^. Typically, SPEs are quantitatively described by their fluence (that is, number of incident particles per cm^2^) of protons with kinetic energies above 30 MeV. It is estimated that the Carrington event was characterized by a fluence ≥30 MeV of 1.9 × 10^10^ protons per cm^2^ (ref. 7). However, this estimate of the Carrington solar flare is debated^8^.

Even though observational advances in the past decades helped to constrain the Sun's eruptive limits, the record is not long enough to assess the frequency of rare extreme flares as have been observed in solar-type stars^9^. A long-term perspective on frequency, fluence and energy distribution of SPEs can be provided by cosmogenic radionuclides^1^ such as beryllium-10, carbon-14 and chlorine-36 (respectively ^10^Be, ^14^C and ^36^Cl) which all arise from the nuclear cascade triggered when cosmic rays reach the atmosphere. Their main production component comes from the incoming galactic cosmic rays as they have, on average, much higher energies than solar particles. The Earth is partially shielded from galactic cosmic rays by the heliomagnetic and the geomagnetic fields, the strengths of which vary from decadal to millennial timescales^10^,^11^. Nevertheless, large outbursts of solar protons can lead to a rapid increase in the atmospheric production of radionuclides which are subsequently stored in environmental archives such as tree rings and ice cores.

Miyake *et al*.^12^,^13^ discovered two large natural rapid increases in atmospheric Δ^14^C (^14^C/^12^C ratio corrected for fractionation and decay, relative to a standard) measured in Japanese cedar trees and dated to AD 774/5 and 993/4. The larger of the two increases (AD 775) was characterized by a sharp enhancement in the atmospheric Δ^14^C of 12‰ over 1 year which corresponds to six times the measurement error and which was estimated to be about 20 times larger than changes attributed to ‘ordinary' solar modulation^12^. As a result, the AD 775 and 994 rapid increases in radiocarbon were linked to exceptional cosmic-ray events which have no counterpart in the instrumental records. A number of potential causes for the AD 775 event have been invoked including a gamma-ray burst^14^,^15^, a cometary event^16^ or a solar proton event^13^,^17^,^18^. Considering that no SPEs including the Carrington solar flare yielded a notable increase in atmospheric radiocarbon concentrations^19^, the magnitude of both the hypothesized AD 775 and 994 solar proton events would have been exceptional. Previous studies^18^,^19^,^20^ have aimed at estimating the possible fluence of the AD 775 event with results varying by as much as two orders of magnitude. This is partly due to different assumptions concerning the energy spectrum of the incident particles.

Here we include new and annually resolved measurements of ^10^Be from three ice cores—the North Greenland Ice core Project (NGRIP), the North Greenland Eemian Ice Drilling (NEEM-2011-S1; henceforth NEEM) as well as the Western Antarctic Ice Sheet Divide Core (WDC) in addition to lower-resolved ^36^Cl data from the Greenland Ice core Project (GRIP)^21^. These radionuclides are produced through different reaction pathways which have different energy dependencies (Fig. 1). This distinguishing feature allows us to better constrain the cause of the events, down to a solar proton event. We further compare the different peaks in production of ^10^Be and ^36^Cl to show that the solar proton events in question were both characterized by a very hard fluence spectrum, with high fluxes of protons with kinetic energies ≥100 MeV. From this, we can deduce that these events were significantly larger than any solar proton event recorded during the satellite era.

## Results

### The events shown in ^10^Be and ^36^Cl records

The time series of ^10^Be, ^14^C and ^36^Cl which are studied herein are displayed in Fig. 2a–c and Fig. 3a–c where the NGRIP, NEEM and WDC chronologies were adjusted to fit the ^14^C peaks found by Miyake *et al*.^12^,^13^ (Methods). The natural background level, that is, the contribution of galactic cosmic rays on the production of radionuclides, was established for each record as the average ^10^Be and ^36^Cl flux values as well as the average ^14^C production rate values prior to and following the peaks. The peaks result from a combination of production and deposition effects leading to an apparent temporal broadening of the measured events. We thus assume that the values exceeding 3*σ* of the natural background level around the AD 775 (filled areas in Fig. 2a–c) and AD 994 peaks (filled areas in Fig. 3a–c) are related to the two events. The amplitudes which we inferred from the peak areas above these natural background levels are displayed in Fig. 2d–f and Fig. 3d–f where error bars were calculated in order to take in consideration measurement uncertainties as well as a background variability of 1*σ* (due to noise in the data and the 11-year solar modulation variability).

Our ^10^Be measurements (Figs 2 and 3, and Table 1) show the existence of the AD 774/5 cosmic-ray event in the Arctic NGRIP and NEEM ice cores as well as in the Antarctic WDC ice core with an average flux enhancement of a factor of 3.4±0.3 (total excess flux related to the average annual background flux). We also report the smaller AD 993/4 event in the ^10^Be records from NGRIP and NEEM with an average flux enhancement of 1.2±0.2 times the natural background level. The agreement between our stacked ^10^Be fluxes and modelled ^14^C production rates (Methods) is comparatively good, especially for the AD 774/5 event which exhibits similar peak amplitudes for both radionuclides. We note, however, that the peak amplitudes in ^10^Be and ^14^C differ somewhat at AD 993/4 but agree within the margin of errors (Fig. 3d-e, Table 1). The difference likely is due to small uncertainties in the ^10^Be measurements (as seen in Fig. 3a with a poorer agreement between the NEEM and NGRIP ice-core data) and/or in the modelled ^14^C production rates (Methods). Our ^10^Be records also indicate that the cosmic-ray event around AD 775 was considerably stronger amounting to a threefold multiple of the AD 994 ^10^Be peak, which is consistent with the Δ^14^C measurements from Miyake *et al*.^12^,^13^. The fact that the AD 994 event was weaker leading to a poorer signal-to-noise ratio also could explain the differences between the NGRIP and NEEM ^10^Be and the tree ring ^14^C records for that time period. In general, the agreement with the three ^10^Be series around AD 774/5 is very good albeit displaying a slightly different structure of the peak itself. This is likely to be related to the fact that a constant sampling resolution per depth (as was used for NGRIP) translates into a somewhat variable temporal sampling resolution due to fluctuations in the annual snow accumulation rates and to different sampling in time for the different sites. Our measurements thus provide first unequivocal evidence of a symmetrical production and deposition of ^10^Be at both poles (bipolar symmetry) during the AD 774/5 event.

**Figure 1:**
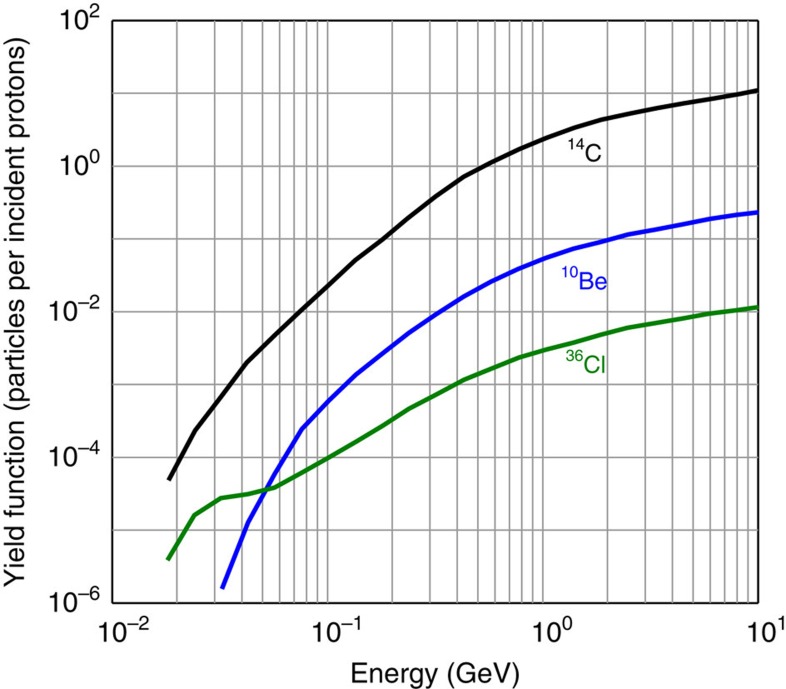
Yield functions of ^10^Be,^14^C, and ^36^Cl. Globally averaged atmospheric production of each radionuclide per unit flux of incident proton, that is, incoming solar cosmic-ray, as a function of kinetic energy. The yield functions are from refs. 35 and 36.

**Figure 2:**
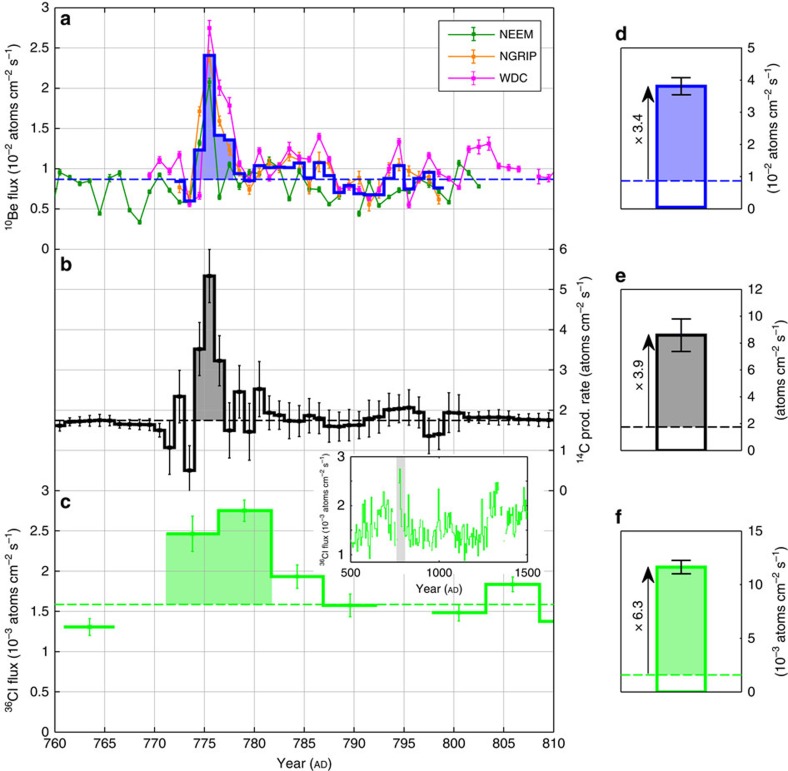
The AD 774/5 event in view of ^10^Be, ^14^C and ^36^Cl. Time series for AD 760–810 (**a**) of ^10^Be flux from the NEEM-2011-S1, NGRIP and WDC ice cores in addition to the inferred average ^10^Be flux (thick blue curve), (**b**) of modelled ^14^C production rate based on previously published measurements^12^ and (**c**) of ^36^Cl flux^21^ in addition to an inset with a longer series spanning AD 500–1500 for ^36^Cl where the grey rectangle represents the time slice investigated. The dashed lines represent the natural background levels which are set as the average values prior to and following the filled areas. The filled areas represent the estimated production enhancements caused by the cosmic-ray event of AD 774/5. The ^10^Be and ^36^Cl series have been corrected for a temporal offset between ice-core and tree-ring chronologies (Methods). The right panel shows radionuclide production enhancements caused by the AD 774/5 event in atoms cm^−2^ s^−1^ for 1 year for (**d**) ^10^Be, (**e**) ^14^C and (**f**) ^36^Cl. The radionuclide increases are illustrated with arrows corresponding to the ratio between the inferred flux/production enhancements stacked over 1 year (coloured rectangles) and the estimated background levels (white rectangles). Uncertainties are based on error propagation including measurement errors and a background variability of 1*σ*.

**Figure 3:**
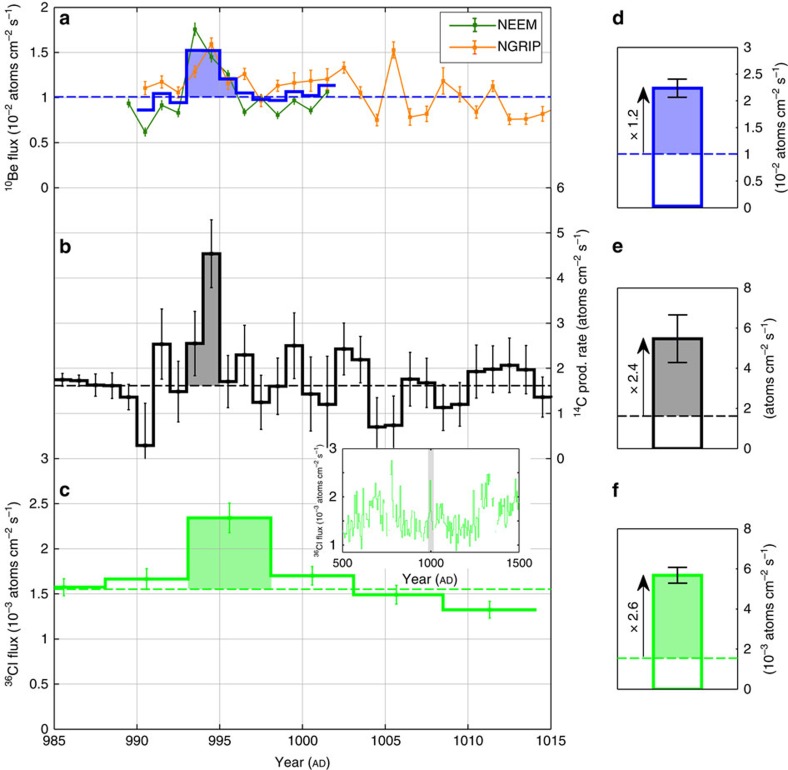
The AD 993/4 event in view of ^10^Be, ^14^C and ^36^Cl. Time series for AD 985–1015 (**a**) of ^10^Be flux from the NEEM-2011-S1 and NGRIP ice cores in addition to the inferred average ^10^Be flux (thick blue curve), (**b**) of modelled ^14^C production rate based on previously published measurements^13^ and (**c**) of ^36^Cl flux^21^ in addition to an inset with a longer series spanning AD 500–1500 for ^36^Cl where the grey rectangle represents the time slice investigated. The dashed lines represent the natural background level which is set as the average value prior to and following the filled areas. The filled areas represent the estimated production enhancement caused by the cosmic-ray event of AD 993/4. The ^10^Be and ^36^Cl series have been corrected for a temporal offset between ice-core and tree-ring chronologies (Methods). The right panel shows radionuclide production enhancements caused by the AD 993/4 event in atoms cm^−2^ s^−1^ for 1 year for (**d**) ^10^Be, (**e**) ^14^C and (**f**) ^36^Cl. The radionuclide increases are illustrated with arrows corresponding to the ratio between the inferred flux/production enhancements stacked over 1 year (coloured rectangles) and the estimated background levels (white rectangles). Uncertainties are based on error propagation including measurement errors and background variability of 1*σ*.

The fact that the ^10^Be and ^14^C increases are imprinted over a time span of 2–3 years despite the probable ephemeral aspect of the cosmic-ray events can be explained by the duration of the transport of the radionuclides from the stratosphere, where they are mostly produced^22^,^23^, to the ground. A smaller fraction of radionuclides also can be produced in the troposphere, which would deposit more rapidly than stratospheric counterparts, given cosmic rays that are sufficiently energetic. The induced complex transport^24^,^25^ of ^10^Be, ^14^C and ^36^Cl could thus lead to a temporal broadening of the deposition peaks. Also, deposition fluxes at a specific ice-core site can be differently impacted by the involved scavenging processes (for example, proportion of wet and dry deposition) and by atmospheric circulation^26^. We here assume that the relative peak amplitudes at the different sites investigated are not affected by such system effects as supported by the comparable average ^10^Be flux and similar relative increases at all three locations (Fig. 2a). In addition, ^36^Cl can be characterized by some mobility in the snowpack^27^ as it is deposited to ice caps in the form of gaseous HCl. Although this is not expected to be a major source of uncertainty at high accumulation sites^27^, this in turn implies that outgassing and migration of ^36^Cl to upper snow layers can occur, further broadening the signal. The ^36^Cl data from the GRIP ice core should consequently be regarded as more uncertain. Nevertheless, there are two large peaks present around AD 775 and 994 which are likely to be attributable to the two cosmic-ray events. The time profile insets in Fig. 2c and Fig. 3c, which span from AD 500 to 1500, emphasize that the two peaks represent the two most conspicuous features of the record during this time slice with flux enhancement factors of 6.3±0.4 and 2.7±0.3 for the AD 774/5 and 993/4 events, respectively. The estimates of the production increases in ^10^Be, ^14^C and ^36^Cl are listed in Table 1. Based on our measured ^10^Be data, modelled ^14^C production rates and to the lower-resolved ^36^Cl data, the production of ^36^Cl was the most enhanced during the two cosmic-ray events in accordance with the expectations for lower energy particles relative to galactic cosmic rays (Fig. 1).

### Supporting a solar origin

It was recently suggested that the radiocarbon peak measured at AD 774/5 was caused by cometary dust from the collision of a bolide into the atmosphere^16^. The authors report a large increase in ^14^C content in corals from the South China Sea around AD 773. They also note that their measurements are coeval with sightings of a comet and dust event documented in ancient Chinese chronicles. However, it was concluded in other studies that the dimensions needed for a comet to account for this additional injection of radiocarbon would need to be significantly more massive^28^,^29^ than the previous estimates^16^. In consequence, the comet would inevitably have had a considerable and observable impact on the geobiosphere. More problematic for the comet hypothesis is that ^10^Be and ^14^C fallouts released from a comet disintegrating in the atmosphere would be, at most, hemispherically redistributed so that the event would only be recorded in either one of the hemispheres^30^. The ^36^Cl peaks arising from the French nuclear bomb tests which mainly occurred around the 1960s represent a good analogy to this. The related ^36^Cl fluxes are significantly larger in the southern hemisphere, where the bomb tests had been undertaken^31^. Moreover, the fact that the peaks around AD 774/5 and 993/4 are reported around the globe and in a multitude of radionuclide records^12^,^13^,^16^,^18^,^30^,^32^,^33^ in addition to their large amplitude is indicative of an enhanced atmospheric production triggered by an extraordinary influx of cosmic rays in both hemispheres.

It was also suggested that a typical signature of a gamma-ray burst (GRB) on the production of different radionuclides, its ‘isotopic footprint', would be a distinct increase in ^14^C and ^36^Cl but not in ^10^Be content^15^. As stated by the authors, the induced secondary neutrons would be at an insufficient energy to initiate spallation reactions on oxygen nuclei and produce detectable amounts of ^10^Be. Thus, a GRB is inconsistent as a possible astrophysical source for the two events in perspective of our newly obtained ^10^Be records (Figs 2 and 3). In addition, the two events are rather similar in that they produced abnormal quantities of ^10^Be, ^14^C and ^36^Cl. This leads us to believe that they share the same cause. In purely probabilistic terms, two GRBs striking the Earth within 200 years is unlikely considering the suggested rate of 1 GRB directed at Earth from our galaxy every 125,000 years^34^. Another diagnostic feature is the above-mentioned bipolar symmetry in the production of ^10^Be, but also in the production of ^14^C (ref. 30). This implies that the incoming particles must have been affected and redirected by the geomagnetic field and, thus, that they were charged. This rules out gamma rays (photons) as triggers of the ^10^Be, ^14^C and ^36^Cl peaks at AD 774/5 and 993/4.

Our data, therefore, support the hypothesis that one or several extreme solar proton events are responsible for the radionuclide production peaks measured at AD 774/5 and 993/4 as it is the only option which is in agreement with all available data. Furthermore, the fact that the ^36^Cl peaks exhibit the largest amplitude mirroring the resonance effect^35^ shown in Fig. 1 constitutes further evidence for a solar origin, that is, being caused by solar cosmic rays which generally have lower energies than galactic cosmic rays.

### Spectral hardness of the SPEs

**Figure 4:**
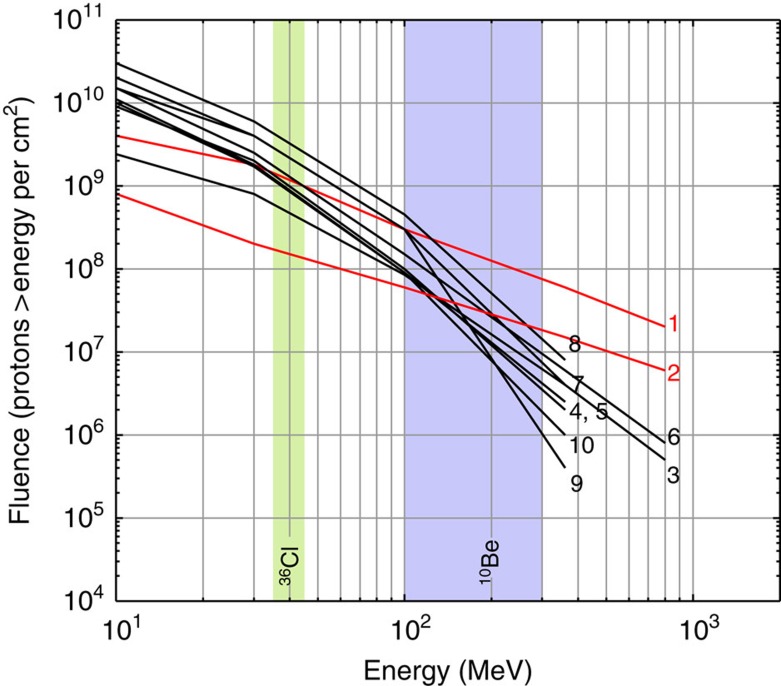
Event-integrated fluence spectra of recorded SPEs. Integral fluence spectra for 10 notable solar proton events which occurred between 1956 and 2005 (ref. 35). The numbers of the spectra relate to Table 2. The green and blue bands represent the approximate specific peak response energies of ^36^Cl and ^10^Be, that is, the incident proton energies at which each radionuclide is mainly produced. The red curves emphasize very hard spectra here defined as leading to a Ground Level Enhancement peak intensity above 1,000% of neutron monitor at sea level on the polar plateau. Modified from ref. 35.

The conclusion that one SPE (or a series of SPEs) is responsible for the production increase of ^10^Be, ^14^C and ^36^Cl at AD 774/5 (Fig. 2) is of particular significance because it implies that it must have reached an exceptional magnitude. In fact, no solar phenomena, including the Carrington event, have ever been unequivocally associated with a distinct increase in ^10^Be concentrations in ice cores. Knowledge of the characteristics of this major solar event, such as its spectral hardness and its fluence ≥30 MeV, could consequently help to better estimate the upper limit of the magnitudes of SPEs. The proton fluences of energy ≥30 MeV, or *F*_30_ of an SPE required to yield a given increase in the production rate of a given radionuclide is directly bound to the spectral hardness of the SPE (that is, the proportion of high energy protons compared to low energy protons). For instance, Webber *et al*.^35^ have listed the *F*_30_ of observed SPEs and computed estimates of their impact on the atmospheric production of ^10^Be and ^36^Cl. They show that the very hard SPE of February 1956 (SPE56), with a *F*_30_ of about 1.8 × 10^9^ protons per cm^2^ yielded five times more ^10^Be than the very soft SPE of August 1972 (SPE72) which yet had a *F*_30_ twice as large. Hence it is crucial to ascertain the spectral hardness of the SPEs around AD 775 and 994 in order to reliably evaluate their *F*_30_. To achieve this, one can exploit the different energy sensitivities of the production rates of cosmogenic radionuclides. For instance, the yield functions of ^10^Be and ^36^Cl have very different shapes at low energies (Fig. 1). As such, the production of solar-induced ^36^Cl nuclides is relatively more sensitive to incident protons at about 30 MeV while the production of ^10^Be nuclides is, compared to ^36^Cl, more sensitive to solar protons at about 100 MeV (ref. 35). A small ratio of the relative production enhancement of ^36^Cl relative to ^10^Be (^36^Cl/^10^Be) would therefore be expected to be indicative of hard SPEs which are characterized by larger amounts of protons ≥100 MeV resulting in a flatter spectrum and vice versa for soft SPEs. As a test, we investigated the relative ^36^Cl/^10^Be ratios of notable SPEs which occurred between 1956 and 2005, for which the spectral characteristics are known and for which ^10^Be and ^36^Cl production yields have been computed^35^. The results are listed in Table 2 while the integral fluence spectra of the related solar proton events are plotted in Fig. 4.

A clear pattern arises from Table 2 with harder SPEs (for example, 1956 and 2005) resulting in lower ^36^Cl/^10^Be production rate ratios. Inversely, softer SPEs (for example, 1959, 1960, 1972 and 2001) show ratios consistently above 3. By using the different energy dependencies of ^10^Be and ^36^Cl and comparing their individual production enhancements, it is thus possible to estimate the spectral shape of paleo-SPEs. From our results, we find the ^36^Cl/^10^Be ratio to be of about 1.8±0.2 and 2.1±0.4 for the SPEs of AD 774/5 and 993/4, respectively. Despite the uncertainties engendered by the ^36^Cl record as described earlier, the ratios put the two solar events in the hard spectrum category. By comparing these ratios to Table 2, we find that the best modern analogue would be a very hard spectrum similar to that of the SPE of January 2005 (SPE05). This result strengthens SPEs as the cause for the events because it rules out the extremely high *F*_30_ estimates, based on softer spectra (for example, SPEs of August 1972 and October 1989), required to yield the ^14^C peak of AD 774/5 and which were associated with moderate to high risks for erythema induced by increased ultraviolet radiation^20^.

## Discussion

**Figure 5:**
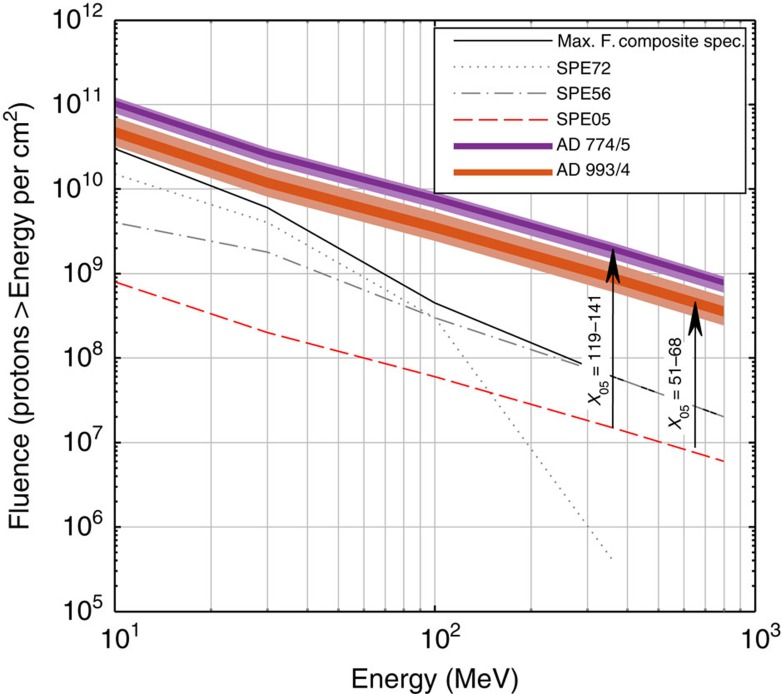
Event-integrated fluence spectra of the AD 774/5 and 993/4 events. Estimated fluence spectra of the extreme SPEs associated with the AD 774/5 and 993/4 events based on ^10^Be and ^14^C and based on the fluence spectrum of SPE05 (red dashed curve) as per ref. 35. The arrows show the scaling factors that were needed to produce the measured ^10^Be and ^14^C when assuming a spectral hardness as per SPE05. For perspective, fluence spectra of a very hard (SPE56) and a soft (SPE72) SPE that occurred during the instrumental era are plotted. The black curve represents a composite series of the highest fluences recorded between 1956 and 2005 for protons at *E*>10, 30, 100, 360 and 800 MeV based on previously published data^35^.

By knowing the approximate spectral hardness of the solar proton events, the specific yield function of ^10^Be as well as the averaged ^10^Be flux from the NGRIP, NEEM and WDC ice cores, we can estimate the fluence. More precisely, we applied the multiple of the ^10^Be increase factor attributable to the SPEs of AD 774/5 and 993/4 (Figs 2d–f and 3d–f and Table 1) relative to that of SPE05 (*X*_*05*_), to the fluence spectrum of the latter (spectrum 2 in Fig. 4). Previous computations^35^ infer that SPE05 caused an annual ^10^Be production increase by a factor of about 0.024 under the assumption that complete atmospheric mixing would take place before deposition. This scenario is more realistic than no mixing at all because solar protons mostly would produce ^10^Be and other radionuclides in the stratosphere which ensures a homogeneous distribution of the cosmogenic radionuclide signature due to a relatively longer mean residence time as opposed to the troposphere^25^. In comparison, our ^10^Be measurements indicate increase factors of 3.4 and 1.2 (Figs 2d–f and 3d–f) implying a multiple *X*_*05*_ of 141 and 51 for the SPEs of AD 774/5 and 993/4, respectively. With a spectral hardness similar to that of SPE05, we therefore find a *F*_30_ of 2.82±0.25 × 10^10^ protons per cm^2^ for the SPE(s) of AD 774/5 and of 1.02±0.21 × 10^10^ protons per cm^2^ for the SPE(s) of AD 993/4 (Fig. 5). In addition, we performed an alternative estimation by multiplying the specific yield function of ^14^C (Fig. 1)^36^ by a scaled up differential energy spectrum of SPE05 (ref. 37) which could account for the amount of ^14^C produced during the two events. A multiple *X*_*05*_ of 119 and 68 was then needed to reproduce the same amount of radiocarbon than modelled which indicates a *F*_30_ of 2.38±0.40 × 10^10^ and 1.36±0.42 × 10^10^ protons per cm^2^ for the SPE(s) of AD 774/5 and 993/4, respectively (Fig. 5). We also performed the same calculation but using a different ^14^C yield function^38^ in order to assess the dependence of our results on the production rate models considered. We found very similar *F*_30_ estimates of 2.76±0.46 × 10^10^ protons per cm^2^ (AD 774/5) and of 1.56±0.48 × 10^10^ protons per cm^2^ (AD 993/4).

Of course, estimating the fluence ≥30 MeV is linked to several assumptions and uncertainties which are difficult to quantify such as the noise inherent to radionuclide data, the true annually resolved ^36^Cl peaks, the carbon cycle modelling (Methods) as well as the yield functions used. However, the very good agreement between our alternative estimates of the *F*_30_ for both events based on different and independent methods supports that the assumptions and uncertainties regarding our ^10^Be fluxes and ^14^C production rates do not affect our findings significantly. As such, the large amplitudes of the peaks measured in a variety of independent records all are indicative of extreme solar events with fluences ≥30 MeV in the order of 10^10^ protons per cm^2^ (Fig. 5). Furthermore, the use of two different ^14^C yield functions^36^,^38^ returned very similar results. In Fig. 5, possible fluence spectra of both events based on the spectrum of SPE05 and on our different estimates of the *F*_30_ are shown with an envelope representing the uncertainties described above. It emphasizes that the *F*_30_ of the SPE(s) at AD 774/5 most likely was well above 10^10^ protons per cm^2^, even considering the lowest bound of our error margin.

Although our *F*_30_ for the larger of the two events (AD 774/5) is slightly lower than previously suggested, based on a spectrum assumed to be similar to that of SPE56 (refs 18, 20), it remains extremely high and unprecedented. Figure 5 also shows that, for both events, the fluence above any given kinetic energy is much larger than for the soft SPE of August 1972 (dotted curve) and for the hard SPE of February 1956 (dashed dotted curve). The same holds true for a composite spectrum encompassing the highest fluence from the SPEs listed in ref. 35 at kinetic energies above 10, 30, 100, 360 and 800 MeV. In a modern context, such magnitudes would most likely lead to important disruptions of satellite-based technologies and means of communication. In addition, the brief but extensive ozone depletion which would follow^20^ could potentially have effects on atmospheric temperatures and circulation.

In conclusion, we have shown that our annually resolved ^10^Be measurements rule out all suggested sources but, and thereby confirm, a solar cause for the AD 774/5 and 993/4 events. We have also shown that the associated solar proton events were most likely characterized by a very hard spectrum. We conjecture that the strongest event (AD 775) had a fluence above 30 MeV at least five times larger than any observed SPE during the instrumental period between 1956 and 2005. This and previous studies should thus motivate the investigation of similar events in order to better ascertain the occurrence rate of severe solar events.

## Methods

### ^10^Be data

Beryllium-10 is available at quasi-biennial resolution from the GRIP although the record is presently not continuous^39^,^40^. Examining the data around AD 775 and 994, it is likely that the events are not yet measured in the record. In addition, ^10^Be data exist from the Dome Fuji ice core in Antarctica^41^ but at a low resolution of 6–15 years. Recently, Miyake *et al*.^33^ performed quasi-annual ^10^Be measurements on the Dome Fuji ice core though the authors report that the record is affected by multiannual climatic variability. To increase the amount of highly resolved data and decrease the impact of noise on the ^10^Be data, we conducted new ^10^Be measurements with annual resolution at depths comprising the two peaks on 3 different ice cores—the NGRIP, the NEEM-2011-S1 as well as the WDC. The NGRIP ice was sampled at a constant spatial resolution of 18.3 cm, resulting in an average temporal resolution of about 1 year. The ^10^Be nuclides were extracted from the ice samples following the chemical procedure described in Adolphi *et al*.^42^, and measured at the Tandem Laboratory in Uppsala, Sweden. Annual NEEM and WDC ^10^Be concentrations were determined according to Woodruff *et al*.^43^ and Sigl *et al*.^44^ and measured at the PRIME laboratory in Purdue, USA. Since radionuclides in ice cores are measured as concentrations, deposition fluxes of ^10^Be were inferred as they might more accurately reflect the production signal by correcting for effects of varying ice accumulation rate^45^. Fluxes also provide a better quantitative estimate of how many atoms were produced and deposited by the AD 774/5 and 993/4 events which can then be compared with computations of the production of ^10^Be nuclides induced by observed solar proton events.

### Timescale adjustments

Age models of ice cores are subject to accumulating uncertainty from the interpretation of ambiguous signatures used for annual layer dating^46^, whereas tree ring chronologies have proven calendar-age accuracy^47^ (at these timescales). The measurement of the ^10^Be peaks unravelled a mismatch between ice-core chronologies and tree-ring timescales of 7 years around AD 775 and 994 (ref. 44), which was attributed to a dating bias in previous ice-core chronologies. In consequence, we adjusted the ^10^Be and ^36^Cl ice-core records to fit the tree rings ^14^C peaks, in agreement with the revised ice-core chronologies proposed for WDC, NEEM and NGRIP^44^.

### ^14^C production rates

In addition to ^10^Be, we utilized the tree rings Δ^14^C data from Miyake *et al*.^12^,^13^ from which the production rates were derived in order to correct for post-production effects linked to the carbon cycle^48^. Radiocarbon content as retrieved from natural archives does not mirror the atmospheric production rate but rather a damped^49^ and time-shifted signal of it due the large active ^14^C reservoirs in the atmosphere, biosphere and ocean and their interactions. We therefore used a carbon cycle box-diffusion model^50^ to rectify this system bias and infer the ^14^C production signal which can explain the atmospheric ^14^C concentration, Δ^14^C (ref. 11). As the model returns normalized production rates, we multiplied the output results by 1.8 atoms cm^−2^ s^−1^. This corresponds to an average value of different suggestions for the mean global pre-industrial production rate of ^14^C ranging from 1.6 (ref. 51) to 2.02 atoms cm^−2^ s^−1^ (ref. 23). The applied carbon cycle model is not optimized for short timescales (that is, years). In consequence, it might not accurately reproduce the shape of the ^14^C increase. Nevertheless, the estimated peak amplitudes should be robust as the model reproduces the integrated longer-term atmospheric ^14^C signal. In comparison, our estimate for the ^14^C production rate induced by the AD 774/5 event (6.84 atoms cm^−2^ s^−1^) is very similar to the findings of Güttler *et al*.^32^ (6.9 atoms cm^−2^ s^−1^).

### ^36^Cl data

Finally, we assessed the existence and amplitude of the peaks in the ^36^Cl data from the GRIP ice core^21^, which has an average uncertainty of 7%, despite its resolution of *circa* 5 years only. This radionuclide is of particular interest because its production rate is relatively more sensitive to protons with energies below 100 MeV (Fig. 1) compared with other radionuclides. More specifically, its yield function shows an excess for protons with energies around 30 MeV attributed to resonances for ^36^Cl production arising from the interaction with atmospheric Ar (ref. 35). Similarly to ^10^Be, deposition fluxes of ^36^Cl were calculated.

## Additional information

**How to cite this article:** Mekhaldi, F. *et al*. Multiradionuclide evidence for the solar origin of the cosmic-ray events of AD 774/5 and 993/4. *Nat. Commun.* 6:8611 doi: 10.1038/ncomms9611 (2015).

## Figures and Tables

**Table 1:** Summary of results.

	^10^Be	^14^C	^36^Cl
*AD 774/5 event*			
Peak factor	3.4±0.3	3.9±0.7	6.3±0.4
Flux enhancement (atoms cm^−2^ s^−1^)	2.94±0.27 × 10^−2^	6.84±1.16	10±0.6 × 10^−3^
			
*AD 993/4 event*			
Peak factor	1.2±0.2	2.4±0.7	2.6±0.3
Flux enhancement (atoms cm^−2^ s^−1^)	1.23±0.17 × 10^−2^	3.86±1.18	4.1±0.4 × 10^−3^

Estimates of the annual production/deposition enhancements of ^10^Be, ^14^C and ^36^Cl caused by the cosmic-ray events of AD 774/5 and 993/4.

**Table 2:** Relative ^36^Cl/^10^Be ratios.

Solar proton event		Relative ^36^Cl/^10^Be ratio
1	23 February 1956	1.2
2	20 January 2005	1.5
	AD 774/5	1.8±0.2
	AD 993/4	2.1±0.4
3	29 September 1989	2.5
4	29 October 2003	3
5	14 July 2000	3.5
6	19 October 1989	3.6
7	10 July 1959	4
8	12 November 1960	4
9	04 August 1972	6
10	04 November 2001	6

The ratios are based on computations of the annual mean production of ^10^Be and ^36^Cl by 10 large solar proton events between 1956 and 2005 (ref. 35). The ratios calculated in this study for the AD 774/5 and 993/4 events are also included. The table is sorted by ascending ratios.

## References

[b1] Schrijver C. . Estimating the frequency of extremely energetic solar events, based on solar, stellar, lunar, and terrestrial records. J. Geophys. Res. 117, A08103 (2012).

[b2] Shea M. & Smart D. Space weather and the ground-level solar proton events of the 23rd solar cycle. Space Sci. Rev. 171, 161–188 (2012).

[b3] Seppälä A. . Solar proton events of October–November 2003: ozone depletion in the Northern Hemisphere polar winter as seen by GOMOS/Envisat. Geophys. Res. Lett. 31, L19107 (2004).

[b4] Lopez-Puertas M. . Observation of NO*x* enhancement and ozone depletion in the Northern and Southern Hemispheres after the October–November 2003 solar proton events. J. Geophys. Res. 110, A09S43 (2005).

[b5] Calisto M., Usoskin I. & Rozanov E. Influence of a Carrington-like event on the atmospheric chemistry, temperature and dynamics: revised. Environ. Res. Lett. 8, 045010 (2013).

[b6] Boteler D. The super storms of August/September 1859 and their effects on the telegraph system. Adv. Space Res. 38, 159–172 (2006).

[b7] Smart D., Shea M. & McCracken K. The Carrington event: Possible solar proton intensity–time profile. Adv. Space Res. 38, 215–225 (2006).

[b8] Wolff E. . The Carrington event not observed in most ice core nitrate records. Geophys. Res. Lett. 39, L08503 (2012).

[b9] Maehara H. . Superflares on solar-type stars. Nature 485, 478–481 (2012). 22622572 10.1038/nature11063

[b10] Beer J. . Information on past solar activity and geomagnetism from 10Be in the Camp Century ice core. Nature 331, 675–679 (1988).

[b11] Muscheler R. . Solar activity during the last 1000yr inferred from radionuclide records. Quat. Sci. Rev. 26, 82–97 (2007).

[b12] Miyake F., Nagaya K., Masuda K. & Nakamura T. A signature of cosmic-ray increase in AD 774-775 from tree rings in Japan. Nature 486, 240–242 (2012). 22699615 10.1038/nature11123

[b13] Miyake F., Masuda K. & Nakamura T. Another rapid event in the carbon-14 content of tree rings. Nat. Commun. 4, 1748 (2013). 23612289 10.1038/ncomms2783

[b14] Hambaryan V. & Neuhäuser R. A Galactic short gamma-ray burst as cause for the ^14^C peak in AD 774/5. Mon. Not. R. Astron. Soc 430, 32–36 (2013).

[b15] Pavlov A. . AD 775 pulse of cosmogenic radionuclides production as imprint of a Galactic gamma-ray burst. Mon. Not. R. Astron. Soc 435, 2878–2884 (2013).

[b16] Liu Y. . Mysterious abrupt carbon-14 increase in coral contributed by a comet. Sci. Rep. 4, 3728 (2014). 24430984 10.1038/srep03728 PMC3893640

[b17] Melott A. L. & Thomas B. C. Causes of an AD 774-775 ^14^C increase. Nature 491, E1–E2 (2012). 23192153 10.1038/nature11695

[b18] Usoskin I. . The AD775 cosmic event revisited: the Sun is to blame. Astron. Astrophys. 552, L3 (2013).

[b19] Usoskin I. G. & Kovaltsov G. A. Occurrence of extreme solar particle events: assessment from historical proxy data. Astron. J. 757, 92 (2012).

[b20] Thomas B. C., Melott A. L., Arkenberg K. R. & Snyder B. R. Terrestrial effects of possible astrophysical sources of an AD 774-775 increase in ^14^C production. Geophys. Res. Lett. 40, 1237–1240 (2013).

[b21] Wagner G. . Reconstruction of the geomagnetic field between 20 and 60 kyr BP from cosmogenic radionuclides in the GRIP ice core. Nucl. Inst. Meth. Phys. Res. 172, 597–604 (2000).

[b22] Lal D. & Peters B. Cosmic ray produced radioactivity on the Earth. Handb. Phys 46, 551–612 (1967).

[b23] Masarik J. & Beer J. Simulation of particle fluxes and cosmogenic nuclide production in the Earth's atmosphere. J. Geophys. Res. 104, 12099–12111 (1999).

[b24] Stohl A. . Stratosphere-troposphere exchange: A review, and what we have learned from STACCATO. J. Geophys. Res.-Atmos. 108, 8516 (2003).

[b25] Heikkilä U., Beer J. & Feichter J. Meridional transport and deposition of atmospheric ^10^Be. Atmos. Chem. Phys. 9, 515–527 (2009).

[b26] Pedro J. . Evidence for climate modulation of the 10Be solar activity proxy. J. Geophys. Res.-Atmos. 111, D21105 (2006).

[b27] Delmas R. . Bomb-test ^36^Cl measurements in Vostok snow (Antarctica) and the use of ^36^Cl as a dating tool for deep ice cores. Tellus B 56, 492–498 (2004).

[b28] Overholt A. C. & Melott A. L. Cosmogenic nuclide enhancement via deposition from long-period comets as a test of the Younger Dryas impact hypothesis. Earth Planet. Sci. Lett. 377, 55–61 (2013).

[b29] Usoskin I. & Kovaltsov G. A comet could not produce the carbon-14 spike in the 8th century. Icarus (2015) in press).

[b30] Jull A. . Excursions in the ^14^C record at AD 774–775 in tree rings from Russia and America. Geophys. Res. Lett. 41, 3004–3010 (2014).

[b31] Heikkilä U. . ^36^Cl bomb peak: comparison of modeled and measured data. Atmos. Chem. Phys. 9, 4145–4156 (2009).

[b32] Güttler D. . Rapid increase in cosmogenic ^14^C in AD 775 measured in New Zealand kauri trees indicates short-lived increase in ^14^C production spanning both hemispheres. Earth Planet. Sci. Lett. 411, 290–297 (2015).

[b33] Miyake F. . Cosmic ray event of AD 774-775 shown in quasi-annual ^10^Be data from the Antarctic Dome Fuji ice core. Geophys. Res. Lett. 42, 84–89 (2015).

[b34] Melott A. L. & Thomas B. C. Astrophysical ionizing radiation and Earth: a brief review and census of intermittent intense sources. Astrobiology 11, 343–361 (2011). 21545268 10.1089/ast.2010.0603

[b35] Webber W., Higbie P. & McCracken K. Production of the cosmogenic isotopes ^3^H, ^7^Be, ^10^Be, and ^36^Cl in the Earth's atmosphere by solar and galactic cosmic rays. J. Geophys. Res. 112, A10106 (2007).

[b36] Castagnoli G. & Lal D. Solar modulation effects in terrestrial production of carbon-14. Radiocarbon 22, 133–158 (1980).

[b37] Mewaldt R. . in Proceedings of the *29th International Cosmic Ray Conference*, 111–114 (Pune, India, 2005).

[b38] Kovaltsov G. A., Mishev A. & Usoskin I. G. A new model of cosmogenic production of radiocarbon 14 C in the atmosphere. Earth Planet. Sci. Lett. 337, 114–120 (2012).

[b39] Muscheler R., Beer J. & Vonmoos M. Causes and timing of the 8200yr BP event inferred from the comparison of the GRIP ^10^Be and the tree ring Δ^14^C record. Quat. Sci. Rev. 23, 2101–2111 (2004).

[b40] Vonmoos M., Beer J. & Muscheler R. Large variations in Holocene solar activity: Constraints from ^10^Be in the Greenland Ice Core Project ice core. J. Geophys. Res.- Space 111, A10105 (2006).

[b41] Horiuchi K. . Ice core record of ^10^Be over the past millennium from Dome Fuji, Antarctica: a new proxy record of past solar activity and a powerful tool for stratigraphic dating. Quat. Geochronol. 3, 253–261 (2008).

[b42] Adolphi F. . Persistent link between solar activity and Greenland climate during the Last Glacial Maximum. Nat. Geosci. 7, 662–666 (2014).

[b43] Woodruff T. E., Welten K. C., Caffee M. W. & Nishiizumi K. Interlaboratory comparison of ^10^Be concentrations in two ice cores from Central West Antarctica. Nucl. Instrum. Methods B 294, 77–80 (2013).

[b44] Sigl M. . Timing and climate forcing of volcanic eruptions for the past 2,500 years. Nature 523, 543–549 (2015). 26153860 10.1038/nature14565

[b45] Muscheler R., Beer J., Wagner G. & Finkel R. C. Changes in deep-water formation during the Younger Dryas event inferred from ^10^Be and ^14^C records. Nature 408, 567–570 (2000). 11117740 10.1038/35046041

[b46] Rasmussen S. O. . A new Greenland ice core chronology for the last glacial termination. J. Geophys. Res. 111, D06102 (2006).

[b47] Büntgen U. . Extraterrestrial confirmation of tree-ring dating. Nat. Clim. Change 4, 404–405 (2014).

[b48] Muscheler R., Beer J., Kubik P. W. & Synal H.-A. Geomagnetic field intensity during the last 60,000 years based on ^10^Be and ^36^Cl from the Summit ice cores and ^14^C. Quat. Sci. Rev. 24, 1849–1860 (2005).

[b49] Siegenthaler U., Heimann M. & Oeschger H. ^14^C variations caused by changes in the global carbon cycle. Radiocarbon 22, 177–191 (1980).

[b50] Siegenthaler U. Uptake of excess CO_2_ by an outcrop-diffusion model of the ocean. J. Geophys. Res. 88, 3599–3608 (1983).

[b51] Goslar T. Absolute production of radiocarbon and the long-term trend of atmospheric radiocarbon. Radiocarbon 43, 743–749 (2001).

